# Impact of cigarette smoking on gut microbial dysbiosis: a structured literature review

**DOI:** 10.1017/gmb.2024.3

**Published:** 2024-05-02

**Authors:** Emmanuel Edoghogho Imade, Nosa Omoregbe Obayagbona

**Affiliations:** 1 Department of Microbiology, Faculty of Life Sciences, University of Benin, Benin City, Nigeria; 2 Department of Nursing and Community Health, School of Health and Life Sciences, Glasgow Caledonian University, Glasgow, Scotland; 3 Department of Environmental Management and Toxicology, Faculty of Life Sciences, University of Benin, Benin City, Nigeria

**Keywords:** public health implications, PRISMA guidelines, CASP tool, Bacteroidetes, Firmicutes

## Abstract

The gut microbiota (GM) comprises microorganisms in the human gastrointestinal tract (GIT). Lifestyle choices like smoking lead to gut dysbiosis. This review assessed the effect of cigarette smoke (CS) on gut microbial dysbiosis (GMD) in active smokers compared to non-smokers, as well as the resulting public health implications. A comprehensive search was conducted using the Cumulative Index to Nursing and Allied Health Literature (CINAHL), Medline, and PubMed. The search result was reported following the Preferred Reporting Items for Systematic Reviews and Meta-Analyses (PRISMA) 2020 guidelines. The Critical Appraisal Skills Programme (CASP) tool was used to evaluate the recruited studies. There were 468 articles found, with 17 of them qualifying for full-text screening. Five of these studies were included in the review. Smoke harmed gut microbe proportions; smokers had more Bacteroidetes and less Firmicutes than non-smokers, affecting their Firmicutes/Bacteroidetes (Fir/Bac) ratio. This has significant public health implications. Organisms enriched in the smokers such as *Bacteroidales eggerthii (B. eggerthii)*, *Bacteroidales bacterium (B. bacterium)* pH 8*, Ruminococcus bromii (R. bromii)*, and *Ruminococcus albus (R. albus)* were found to be positively correlated with inflammatory biomarkers. Other organisms, such as *Eubacterium eligens (E. eligens), Eubacterium ramulus (E. ramulus), Eubacterium rectale (E. rectale), Eubacterium ventriosum (E. ventriosum)*, *Roseburia hominis (R. hominis), Ruminococcus torques (R. torques),* and *Roseburia inulinivorans (R. inulinivorans),* were negatively correlated with inflammatory markers and were more in non-smokers.

## Introduction

Microorganisms that inhabit the human gastrointestinal tract (GIT) are collectively referred to as the gut microbiota (GM) (Sorboni et al., 2022). These organisms are closely associated with human biology and play a vital role in several body functions, including resistance to the colonization of non-indigenous microorganisms, immune maturation, digestion, and synthesis of essential nutrients (Pickard et al., 2017; Pant et al., 2022). The term “gut dysbiosis” refers to the imbalance of the GM that is associated with a harmful outcome. Berg et al. (2020) defined microbiota as the community of microorganisms inhabiting a particular environment. In contrast, the term “microbiome,” in a broader sense, encompasses not only the microorganisms themselves but also their genetic material and the surrounding environmental conditions (Nazir et al., 2024). Several immune-related neurological illnesses, like neurodegeneration and developmental abnormalities, have been linked to changes in the GM and synthesis of their metabolites (Sittipo et al., 2022).

Microorganisms have maintained symbiosis with the gut environment throughout evolution. The human gut supplies nutrition and a habitat for intestinal bacteria, which in turn helps to ferment carbohydrates and manufacture vitamins by lowering intestinal permeability and boosting the epithelial defence system to create a mucosal barrier (Berg et al., 2020). The gut mucosal immune system is the most powerful immune system in vertebrates and works in close collaboration with the intestinal microorganisms (Garcia-Carbonell et al., 2019). The balance of the intestinal mucosa immune system is crucial to maintain homeostasis and defend the host (Chunxi et al., 2020).

### Healthy gut microbial composition

Over 100,000 billion microorganisms are found in the human GIT, which corresponds to 10–100 times the number of entire human cells (Thursby and Juge, 2017). Although Rinninella et al. (2019) argue that a universally ideal composition of GM does not exist due to individual variations resulting from factors such as the transition from infancy, antibiotic usage, lifestyle, nutritional habits, and cultural practices. Arumugam et al. (2011) assert that Actinobacteria, Bacteroidetes, Firmicutes, Fusobacteria, Proteobacteria, and Verrucomicrobia are the major phyla of gut bacteria, with Firmicutes and Bacteroidetes accounting for 90% of the GM. They further reported that there are more than 200 different genera in the Firmicutes phylum, including Bacillus, Lactobacillus, Clostridium, Ruminococcus, and Enterococcus. The phylum Actinobacteria is proportionately less prevalent and is mostly represented by the genus Bifidobacterium.

The gut microbe balance can be disrupted by a variety of reasons, including modifications in the gut bacteria or in the mucus layer and epithelial damage brought on by lifestyle choices (Mu et al., 2017). As a result, intestinal permeability is raised, and luminal contents are transported to the underlying mucosa. The pathophysiology of numerous GI illnesses, such as viral enterocolitis, small intestine tract overgrowth, irritable bowel syndrome, and allergic food intolerance, has been linked to the dysregulation of any of these components (Fasano, 2012). Recent research has demonstrated a link between gut microbial dysbiosis (GMD) and the aetiology of numerous chronic diseases, including colorectal cancer (CRC) (Fong et al., 2020), metabolic disorders and gastrointestinal dysmotility (Singh et al., 2021), cardiovascular diseases and hypertension (Lau et al., 2017), inflammatory bowel disease (IBD) (Dolan and Chang, 2017), chronic obstructive pulmonary disease (COPD) (Ananya et al., 2021), and type 2 diabetes (T2D) mellitus and obesity (Rastelli et al., 2019).

### Possible processes through which GMD is brought on by tobacco smoking

The deleterious health effects of tobacco, extensively studied through numerous investigations, are primarily associated with systemic pathophysiological changes attributed to its chemical, heavy metal, particulate matter, and microbial constituents (IARC Working Group on the Evaluation of Carcinogenic Risks to Humans, 2004; Rooney et al., 2005; Larsson et al., 2008). Notably, microbial aspects in tobacco have been relatively underexplored in recent years, potentially serving as causative factors in smoking-related diseases (Huang and Shi, 2019). In a study conducted by Sapkota et al. (2010), it was reported that cigarettes manufactured in the European Union were found to contain 15 distinct bacterial classes, showcasing significant bacterial diversity, including potential pathogens such as *Acinetobacter*, *Bacillus, Burkholderia, Clostridium, Klebsiella,* and *Pseudomonas aeruginosa.*

Cigarette smoking influences the GM through multiple avenues, including immune system modifications, biofilm development, and microenvironmental alterations, potentially contributing to diverse diseases. Impaired antimicrobial defences due to the immunosuppressive effects of tobacco, affecting the peripheral immune system, may permit the colonization of novel bacteria (Matthews et al., 2012). Additionally, the smoky environment, resulting from cigarette smoke (CS), might confer metabolic advantages, promoting biofilm formation and enhanced adherence to epithelial surfaces by specific bacterial taxa. Studies suggest that CS-induced biofilm formation could favour microbial colonization and persistence, contributing to infections (Mutepe et al., 2013). The “microenvironment,” encompassing factors like oxygen tension, pH, and acid production, is pertinent to the influence smoking has on microbiota members. Current smokers exhibit alterations in the upper GIT, including changes in bacterial abundance associated with oxygen tension variations. Consequently, changes in duodenal bicarbonate secretion and lower pH in smokers may exert selective pressure on specific bacterial taxa (Mason et al., 2015; Ganesan et al., 2017).

### Benefits of the GM

The GM confer myriads of benefits to the host, including production of different vitamins, antimicrobial peptides, biotransformation of bile, and synthesis of all essential and non-essential amino acids (Vyas and Ranganathan, 2012; Imade et al., 2021). The formation and operation of immune cells such as T cells, natural killer cells, dendritic cells, macrophages, and invariant natural killer T (iNKT) cells depend critically on the GM (Liu et al., 2013). Moreover, the production of short-chain fatty acids (SCFAs), the regulation of systemic inflammation, and the development of oral immunological tolerance via regulatory T cells (Tregs) are all potential ways that the GM contribute to and maintain body homeostasis (Samuelson et al., 2015). Pais et al. (2020) reaffirmed that they modulate host protection and immune system development through a mechanism known as the competitive exclusion or barrier effect, while Ma et al. (2019) emphasized that they can affect the pharmacological response to medications. In addition, it has been suggested that restoring GM balance can prevent or cure muscle loss due to neuromuscular diseases or ageing (Gizard et al., 2020).

### Identification of gaps in knowledge and justification for study

The unique composition of the gut bacterial population in the colon and stomach is influenced by physicochemical parameters like intestinal motility, pH level, nutrition, and host secretions (digestive enzymes, gastric acid, mucus, and bile) (Zhang et al., 2015). Madore et al. (2020) further elaborated that a variety of factors, such as antibiotic use, stress, ageing, illness, poor diet, and lifestyle choices such as cigarette smoking, could influence GM. Among these factors, cigarette smoking has been reported to be the primary cause of cancer and COPD (Gui et al., 2021).

Numerous quantitative studies have now examined the effect of CS on GM composition in active smokers as compared to non-smokers. Previous reviews have summarized these results in healthy adults (Antinozzi et al., 2022) and in connection to the molecular interaction between CS and GMD (Gui et al., 2021). Numerous new studies have been published in this field since these reviews were written. This is a result of the rapidly expanding body of research on GM, which necessitates an updated synthesis. This review aims to synthesize the most recent data on the effect of CS on GMD in active smokers relative to non-smokers, as well as the resulting public health implications.

## Methods

An extensive search of the Cumulative Index to Nursing and Allied Health Literature (CINAHL), Medline, PubMed, and Google Scholar was conducted to identify studies addressing the effect of tobacco smoke on the composition of GM. Medline is an excellent resource for journal articles in the biomedical as well as life sciences, whereas Cochrane is a collection of six databases containing various forms of high-quality, independent evidence that can also assist in guiding healthcare choices. PubMed is a huge resource with over 5600 journals indexed biomedical and life sciences database maintained by the National Center for Biotechnology Information (NCBI). Additionally, CINAHL indexes materials from the majority of notable nursing groups and other reputable publishers (Haby et al., 2016). These databases were selected because they implement a more systematic approach compared to Google Scholar searches. By combining search topics, employing alternative terms and phrases, filtering, limiting, and saving search results, users can discover information more efficiently and quickly.

As shown in Table 1, appropriate subject headings or key phrase components of the research frame were identified to begin with. These queries were recorded using the Boolean operator “OR,” and they comprised the first hits (S1). In addition, the terms (tobacco OR cigarette OR nicotine OR smok*) AND (microbial OR microflora OR flora OR microbio* OR bacteria*) AND (gut OR intestinal) were inputted using the specified truncations, Boolean operators, asterisks, and inverted commas. The second hits (S2) were derived from these search results. Following that, using the Boolean operator “AND,” the first hit (S1) and the second hit (S2) were linked (S1 AND S2). This resulted in a final list of hits containing all potentially relevant articles identified with the subject headers or containing the key phrases and key terms. This search strategy is illustrated in Table 1. RefWorks was used to store/organize the research, integrate the citations, and build the reference list of works cited.

**Table 1.** Search procedure using key phrases and keywords

Search tool		Search results
PICO framework	Key phrases	S1
Population	“Cigarette smoker” OR “Tobacco smoker”	
Intervention	“Cigarette smoke” OR “Tobacco smoke”	
Control	“Non–smoker”	
Outcome	“Gut dysbiosis” OR “gut microbiota” OR “Gut microbiome” OR “intestinal micro*”	
S2		
Boolean operators	Key words	Search results
	Cigarette OR tobacco OR smok* OR nicotine	S2
AND	Microb* OR bacteria* OR flora OR microflora	
AND	Gut OR intestinal OR dysbiosis	
S3		
Boolean operators	Search tool	Search result
OR	(S1 AND S2)	S3

### Inclusion and exclusion criteria

**Figure 1.**
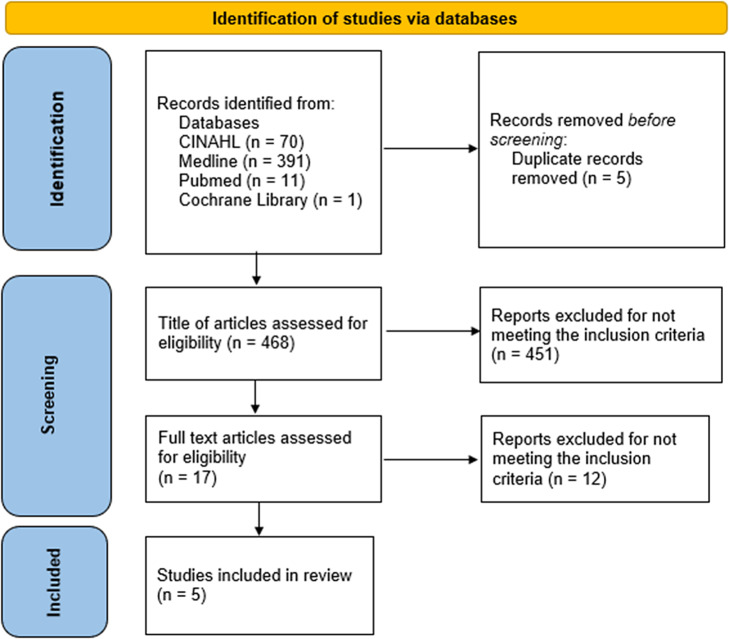
PRISMA flow chart showing the selection process of included studies from database search.

This review considered only peer-reviewed articles of primary studies that examined the effect of CS on GMD in human subjects or the corresponding health outcome. The assessment was conducted according to the quality evaluation procedure outlined in Section “Quality assessment.” To obtain recent findings while avoiding the rigours of interpretation, date and language of publication limitations were implemented. The search was limited to publications published in English between 2016 and 2023. The information flow from selected databases to studies included in the quantitative synthesis is described using the PRISMA 2020 flow diagram in Figure 1 (Shamseer et al., 2015). This instrument has been used to report on both included and excluded studies.

### Quality assessment

For the purpose of this structured literature review (SLR), the CASP tool for cohort study was used in accordance with the prescribed questions to systematically assess and interpret the primary cohort studies included in this review. The CASP tool has been endorsed by the Cochrane Qualitative and Implementation Methods Group as the most used instrument for quality appraisal in health-related evidence syntheses (Long et al., 2020). It has different specific checklists for randomized controlled trials, qualitative studies, systematic reviews, cohort studies, case–control studies, diagnostic studies, clinical prediction rules, and economic evaluations.

Table 5 provides a detailed analysis of how the CASP tool was utilized in each of the principal investigations. The methodological quality of each study was independently assessed using the established criteria in the CASP tool for cohort studies. Only studies with a high score on the evaluation instrument were considered for review.

## Results

The PRISMA flow diagram that illustrates this research selection procedure is shown in Figure 1. This was adapted from the PRISMA 2020 statement: an updated guideline for reporting systematic reviews (Page et al., 2021).

A total of 70 articles were obtained from CINAHL, 391 from Medline, 11 from PubMed, and only one from Cochrane Library. The inclusion and exclusion criteria informed the initial literature search. This yielded five articles from CINAHL, 14 from Medline, and three from PubMed. Three of the articles obtained from CINAHL were also present in Medline. Furthermore, two articles appeared in CINAHL, Medline, and PubMed, while the article obtained from Cochrane Library was not relevant to the study. A total of 17 articles were left after this stage. The final selection of papers for inclusion in the review was made by examining titles, abstracts, and full texts of papers to determine which met the inclusion/exclusion criteria and could provide answers to the research questions. After a thorough examination of 17 publications, only five were retained.

A tabular representation of the selected articles can be found in Table 2

**Table 2.** Selected studies

S/N	Title	Author	Year	Journal	Volume, issue, and page
1	Association between cigarette smoking status and composition of gut microbiota: population–based cross–sectional study	Lee, S.H., Yun, Y., Kim, S.J., Lee, E.J., Chang, Y., Ryu, S., Shin, H., Kim, H.L., Kim, H.N. and Lee, J.H.	Lee et al. (2018)	Journal of Clinical Medicine	*7*(9), p.282.
2	Effects of tobacco smoke and electronic cigarette vapor exposure on the oral and gut microbiota in humans: a pilot study	Stewart, C.J., Auchtung, T.A., Ajami, N.J., Velasquez, K., Smith, D.P., De La Garza II, R., Salas, R., and Petrosino, J.F.	Stewart et al. (2018)	PeerJ	*6*, p.1–16
3	The association between smoking and gut microbiome in Bangladesh	Nolan–Kenney, R., Wu, F., Hu, J., Yang, L., Kelly, D., Li, H., Jasmine, F., Kibriya, M.G., Parvez, F., Shaheen, I., and Sarwar, G.	Nolan–Kenney et al. (2020)	Nicotine and Tobacco Research	*22*(8), pp.1339–1346
4	The effects of cigarettes and alcohol on intestinal microbiota in healthy men	Lin, R., Zhang, Y., Chen, L., Qi, Y., He, J., Hu, M., Zhang, Y., Fan, L., Yang, T., Wang, L., and Si, M.	Lin et al. (2020)	Journal of Microbiology	*58*, pp.926–937
5	Effects of smoking on inflammatory markers in a healthy population as analyzed via the gut microbiota	Yan, S., Ma, Z., Jiao, M., Wang, Y., Li, A., and Ding, S.	Yan et al. (2021)	Frontiers in Cellular and Infection Microbiology	11, p.1–12

### Characteristics of included studies

#### Study population

The sample size for a study should be determined at the planning stage of a study. Andrade (2020) argues that a sample that is either too large or too small is both unscientific and unethical. The authors of the first empirical study recruited for this review conducted a cohort analysis of Korean men and women who go through medical tests annually or biennially at the Kangbuk Samsung Hospital Healthcare Screening Center, South Korea. There were 758 healthy men, ranging in age from 23 to 78 years, who took part (Lee et al., 2018). The second research likewise enrolled 21 men and 12 women with a mean age of 41.67 ± 11.90 years (Stewart et al., 2018). The presence of any systemic disease (such as diabetes, hypertension); excessive alcohol consumption (more than 25 grams per day for men and more than 15 grams per day for women); use of any of the following medications during the previous month, including antibiotics, antivirals, hypoglycaemic medications, blood pressure-lowering medications, lipid-lowering medications, or stomach medications; and abnormal abdominal ultrasound results were the exclusion criteria.

Another study under consideration involved a prospective cohort study of 250 respondents between 25 and 50 years old and free from any major illness. These individuals were chosen at random from communities located in Araihazar, Bangladesh (Nolan-Kenney et al., 2020). Also under review is a study conducted by Lin et al. (2020) who recruited 116 healthy male subjects and divided them into four groups: Group A (non-smoking and non-drinking), Group B (smoking only), Group C (drinking only), and Group D (smoking and drinking combined). The last study under consideration comprised healthy participants between the ages of 22 and 75 years. Exclusion criteria included the use of probiotics, antibiotics, or proton-pump inhibitors within the previous month; symptoms of heart, kidney, liver, or lung diseases; thyroid disease or diabetes mellitus; and any history of digestive tract-related diseases or surgeries, such as gastrointestinal polyp, gastric ulcer, intestinal adenoma, or gastrointestinal tumours (Yan et al., 2021).

#### Research question/aim

All the studies reviewed in this article aimed to evaluate the connection between smoking and the microbiota of the GIT. There were, however, slight differences such as one which made efforts to eliminate some other factors that affect GM (Lee et al., 2018), exploration of electronic cigarette (EC) vapour and tobacco smoke exposure (Stewart et al., 2018), evaluation of the combined effects of cigarette smoking and alcohol consumption (Lin et al., 2020), and use of whole-genome sequencing (WGS) to explore the effects of smoking on the GM at the species level (Yan et al., 2021).

#### Methods

All studies under review involved the extraction of deoxyribonucleic acid (DNA) from faecal samples using DNA extraction kits. Fresh faecal samples were collected from the subjects, immediately frozen at −20 °C, and were placed at −70 to −80 °C within 24 hours. Fusion primers that targeted the variable V3 and V4 regions of the 16S ribosomal RNA (16S rRNA) gene with indexing barcodes were used to amplify the genomic DNA. Samples were pooled for sequencing on the Illumina MiSeq platform. The merged reads then underwent a quality filter, and reads with more than 0.5% predicted errors were eliminated (Lee et al., 2018; Stewart et al., 2018; Lin et al., 2020; Nolan-Kenney et al., 2020). The standard protocol for DNA extraction and 16S rRNA gene sequencing and phylogenetic classification of the isolates were carefully observed in these studies. The 16S rRNA gene had been an integral component of sequence-based bacterial investigation for decades until the discovery of high-throughput sequencing of the whole gene. In line with this, DNA isolated from stool samples was subjected to shotgun metagenomic sequencing using combined probe-anchoring synthesis by Yan et al. (2021). In addition, the raw sequenced reads were subjected to quality control to eliminate low-quality reads using the overall accuracy (≥0.8) control technique.

Table 3 shows the sample size and country of residence of respondents that were recruited for the studies under review. Also presented in the table are the study design, exclusion criteria and methodology of the studies.

**Table 3.** Methodology of reviewed studies

Study	Sample size	Country	Study design	Exclusion criteria	Methodology
Lee et al. (2018)	CCS (n = 203) FS (n = 267) NS (n = 288)	South Korea	Cross–sectional	Use of antibiotics, probiotics, or cholesterol–lowering medication	16S rRNA gene sequencing from faecal DNA
Stewart et al. (2018)	ECU (n = 10) CS (n = 10) NS (n = 10)	United States	Cross–sectional	Not stated	16S rRNA gene sequencing from faecal DNA
Nolan–Kenney et al. (2020)	CCS (n = 62) FS (n = 36), NS (n = 151)	Bangladesh	Longitudinal study	Antibiotic use in the previous month Willingness to come to the clinic to provide stool samples and complete lifestyle questionnaire	16S rRNA gene sequencing from faecal DNA
Lin et al. (2020)	NSD (n = 14) SO (n = 31) DO (n = 28) SD (n = 43)	China	Cross–sectional study	History of digestive tract–related diseases or surgeries The use of antibiotics, probiotics, or proton pump inhibitors in the past month Evidence of heart, liver, kidney, or lung diseases; thyroid disease; or diabetes mellitus	16S rRNA gene sequencing from faecal DNA
Yan et al. (2021)	CCS (n = 33) NS (n = 121)	China	Cross–sectional study	Any systemic disease (hypertension, diabetes, etc.) Excessive alcohol consumption (>25 grams/day for men and > 15 grams/day for women) Use of any of the following drugs within the previous 6 months: antibiotics, antivirals, hypoglycaemic drugs, blood pressure–lowering drugs, lipid–lowering drugs, or stomach medication An abnormal abdominal ultrasound examination	Shotgun metagenomic sequencing from faecal DNA

Abbreviations: CCS, current cigarette smokers; DO, drinking only; ECU, electronic cigarette users; FS, former smokers; NS, never smoked; NSD, non-smoking and non-drinking; SD, smoking and drinking combined; SO, smoking only.

#### Intervention/exposure

All the studies in this review examined the effect of cigarette on GM using human subjects. Lee et al. (2018) and Yan et al. (2021) examined the effect of only cigarettes, while Stewart et al. (2018), Lin et al. (2020), and Nolan-Kenney et al. (2020) included the effect of EC, bidis (unfiltered locally produced thin cigarettes filled with tobacco and wrapped in leaves), and alcohol, respectively. However, for the purpose of this review, only data obtained from the subjects that took cigarette only were extracted.

The criteria for measuring the level of exposure to CS were presented using standard protocols identified by the various researchers. Lee et al. (2018) divided the respondents into three groups: never smokers, former smokers who smoked 14.5 cigarettes/day but had not smoked cigarette during the preceding six months, and current smokers who took 14.3 cigarettes/day. Inclusion requirements for tobacco users in another study included passing the Fagerstrom test for nicotine dependence 4 and smoking at least 10 cigarettes daily (Stewart et al., 2018). ECU in this study vaped often all day, used ECs every day, and had been using ECs actively for about three years. Nolan-Kenney et al. (2020) recruited married adults that smoked an average of 0.50 ± 0.31 packs of cigarettes/bidis per day and classified them as current smokers. Packs per day were calculated as the number of sticks smoked per day divided by 20. Although Yan et al. (2021) did not state the number of cigarettes/day smoked by the participants, like the other studies, they ensured that the participants were healthy adults.

#### Result of empirical studies

The findings reported from the studies indicated that CS exhibited a negative impact on the relative abundances of gut microorganisms. Generally, higher levels of Bacteroidetes, Prevotella, Erysipelotrichia, Catenibacterium, Coriobacteriia, Collinsella, Slackia, Pseudomonas, Actinomyces, *Lachnospira bacterium (L. bacterium)* 1157FAA, *Ruminococcus albus (R. albus),* and *Ruminococcus bromii* (*R. bromii*) were observed in current smokers. Although there was generally a higher level of Bacteroidetes, Stewart et al. (2018) recorded higher Prevotella and lower Bacteroides both of which belong to the phylum Bacteroidetes. Members of the phylum Firmicutes and genus Phascolarctobacterium were observed to be lower in the stool samples of current smokers. Furthermore, the Firmicutes/Bacteroidetes (Fir/Bac) ratio was lower in current smokers than in non-smokers.

However, for non-smokers, there were higher levels of Firmicutes; Actinobacteria; and species of the genera Alistipes, Bacteroides, Eubacterium, and Roseburia. Members of the phyla Bacteroidetes and Proteobacteria and genera Prevotella, Erysipelotrichia, Catenibacterium, Coriobacteriia, Collinsella, and Slackia were observed to be lower. These findings are presented in Table 4.

**Table 4.** Brief result of empirical studies

Research title	Intervention/exposure	Outcome		References
		Current smokers	Non-smokers	
Association between cigarette smoking status and composition of gut microbiota: population–based cross–sectional study	The current smokers examined took an average of 14.5 sticks of cigarettes/day	Higher Bacteroidetes Lower Firmicutes Lower Fir/Bac ratio	Lower Bacteroidetes Higher Firmicutes Higher Fir/Bac ratio	Lee et al. (2018)
Effects of tobacco smoke and electronic cigarette vapor exposure on the oral and gut microbiota in humans: a pilot study	Fagerstrom test for nicotine dependence ≥4 and smoked a minimum of 10 cigarettes per day	Higher Prevotella and lower Bacteroides	Lower Prevotella and higher Bacteroides	Stewart et al. (2018)
The association between smoking and gut microbiome in Bangladesh	An average of 0.50 ± 0.31 packs of cigarettes.	Higher Erysipelotrichia, Catenibacterium, Coriobacteriia, Collinsella, and Slackia	Lower Erysipelotrichia, Catenibacterium, Coriobacteriia, Collinsella, and Slackia	Nolan–Kenney et al. (2020)
The effects of cigarettes and alcohol on intestinal microbiota in healthy men	Subjects smoked continuously or accumulatively for six months or more in their lifetime	Higher Bacteroidetes, Pseudomonas, and Actinomyces Lower Firmicutes Phascolarctobacterium	Higher Firmicutes and Actinobacteria Lower Bacteroidetes and Proteobacteria	Lin et al. (2020)
Effects of smoking on inflammatory markers in a healthy population as analyzed via the gut microbiota	Participants were drawn from a healthy population that attended a health facility for routine check–up	53 spp. were enriched, including *Bacteroidales bacterium, Bacteroides eggerthii, Bacteroides massiliensis, Lachnospira bacterium* 1157FAA, *Ruminococcus albus,* and *Ruminococcus bromii*	41 spp. were enriched, including *Alistipes finegoldii, Alistipes senegalensis, Bacteroides caccae, Bacteroides cellulosilyticus, Bacteroides intestinalis, Eubacterium eligens,* and *Roseburia hominis*	Yan et al. (2021)

### Result of methodological quality assessment

The CASP (2018) checklists for cohort study for quality assessment were adopted for this research. This is presented in Table 4. This assessment tool takes into consideration three broad issues when appraising a cohort study. These questions include the following: Firstly, are the results of the study valid? Secondly, what are the results? And finally, will the results help locally? The set of questions developed in the CASP tool to help in systematically evaluating these topics is discussed in the next section.

The CASP checklist for cohort study is presented in Table 5.

**Table 5.** CASP checklist for cohort study

Appraisal criteria	Study	Appraisal criteria met?			Comment
		Yes	Cannot tell	No	
Section A:	Are the results of the study valid?				
1. Did the study address a clearly focused issue?	Lee et al. (2018)	*			Each study addressed a distinctly defined issue. The identified population consisted of cigarette smokers, while the control group consisted of non–smokers. The studied risk factor was the effect of CS, and the outcome was intestinal microbial dysbiosis
	Stewart et al. (2018)	*			
	Nolan–Kenney et al. (2020)	*			
	Lin et al. (2020)	*			
	Yan et al. (2021)	*			
2. Was the cohort recruited in an acceptable way?	Lee et al. (2018)	*			There was no selection bias that could compromise the generalizability of the findings, as the recruited cohort was representative of the defined population
	Stewart et al. (2018)	*			
	Nolan–Kenney et al. (2020)	*			
	Lin et al. (2020)	*			
	Yan et al. (2021)	*			
3. Was the exposure accurately measured to minimize bias?	Lee et al. (2018)	*			To avoid measurement or classification bias, the intensity of exposure was measured precisely. The participant smoked 14.3 cigarettes per day (Lee et al., 2018), 10 cigarettes per day (Stewart et al., 2018), and 0.50 0.31 packs of cigarettes/bidis per day (Nolan–Kenney et al., 2020). Although Lin et al. (2020) and Yan et al. (2021) did not specify the exact number of cigarettes smoked per day, they recruited subjects based on the WHO (1998) standard which classifies smokers as those who have smoked continuously or accumulatively for at least six months in their lifespan
	Stewart et al. (2018)	*			
	Nolan–Kenney et al. (2020)	*			
	Lin et al. (2020)		*		
	Yan et al. (2021)		*		
4. Was the outcome accurately measured to minimize bias?	Lee et al. (2018)	*			The outcome was accurately measured by the researchers using valid objective measurement protocols
	Stewart et al. (2018)	*			
	Nolan–Kenney et al. (2020)	*			
	Lin et al. (2020)	*			
	Yan et al. (2021)	*			
5. (a) Have the authors identified all important confounding factors	Lee et al. (2018)_	*			Lee et al. (2018), Stewart et al. (2018), and Yan et al. (2021) identified confounding factors such as the presence of systemic disease, excessive alcohol consumption, and use of specific medications during the previous month. However, Lin et al. (2020) and Nolan–Kenney et al. (2020) did not specify these details precisely
	Stewart et al. (2018)	*			
	Nolan–Kenney et al. (2020)		*		
	Lin et al. (2020)		*		
	Yan et al. (2021)	*			
5. (b) Did they take account of the confounding factors in the design and/or analysis?	Lee et al. (2018)	*			Using exclusion criteria, the authors were able to exclude the confounding variables
	Stewart et al. (2018)	*			
	Nolan–Kenney et al. (2020)	*			
	Lin et al. (2020)	*			
	Yan et al. (2021)	*			
6. Was the follow–up of subjects complete and long enough	Lee et al. (2018)	*			At the time of sampling, the follow–up was complete and lengthy enough because the GM of the subjects had been exposed to CS for at least six months. Therefore, positive or negative effects should have had sufficient time to manifest
	Stewart et al. (2018)	*			
	Nolan–Kenney et al. (2020)	*			
	Lin et al. (2020)	*			
	Yan et al. (2021)	*			
Section B: What are the results?					
7. What are the results of this study?	Lee et al. (2018)	The results reflect the variation in the relative diversity of the GM of cigarette smokers and non–smokers. Higher Bacteroidetes, lower Firmicutes, and lower Fir/Bac ratio were observed for smokers, while the reverse was recorded for non–smokers			
	Stewart et al. (2018)				
	Nolan–Kenney et al. (2020)				
	Lin et al. (2020)				
	Yan et al. (2021)				
8. How precise are the results?	Lee et al. (2018)	The results precisely answer the research objectives. It demonstrates that normal human gastrointestinal microbiota contains fewer Bacteroidetes and more Firmicutes, whereas cigarette smoking increases Bacteroidetes and decreases Firmicutes. It further demonstrates that this modification results in negative public health outcomes, including diabetes, obesity, Crohn’s disease, and compromised immunity			
	Stewart et al. (2018)				
	Nolan–Kenney et al. (2020)				
	Lin *et al.*, 2020				
	Yan et al. (2021)				
9. Do you believe the results?	Lee et al. (2018)	*			The results were obtained using established research design and procedures that eliminated bias and confounding variables
	Stewart et al. (2018)	*			
	Nolan–Kenney et al. (2020)	*			
	Lin et al. (2020)	*			
	Yan et al. (2021)	*			
10. Can the results be applied to the local population?	Lee et al. (2018)	*			The results of these studies can be applied to local populations in various parts of the world because the study cohorts were also drawn from diverse locations
	Stewart et al. (2018)	*			
	Nolan–Kenney et al. (2020)	*			
	Lin et al. (2020)	*			
	Yan et al. (2021)	*			
11. Do the results of this study fit with other available evidence?	Lee et al. (2018)	*			Results from these studies corroborate other evidence available in the scientific literature, including those published by Seksik (2010); Sokol and Halfvarson et al. (2017); Savin et al. (2018); and Hiippala et al. (2020)
	Stewart et al. (2018)	*			
	Nolan–Kenney et al. (2020)	*			
	Lin et al. (2020)	*			
	Yan et al. (2021)	*			
12. What are the implications of this study for practice?	Lee et al. (2018)	*			The review substantially contributes to public health policy and practice by highlighting a key consequence of cigarette smoking. The data from this study can be utilized by policymakers and practitioners to design strategies for educating the public about the effects of smoking on intestinal flora
	Stewart et al. (2018)	*			
	Nolan–Kenney et al. (2020)	*			
	Lin et al. (2020)	*			
	Yan et al. (2021)	*			

Asterisks represent the categories under which each study falls, based on the appraisal criteria; Yes/Cannot tell/No.

#### Did the study address a clearly focused issue?

This outlines the scientific context and justification for the reported investigation. A good research project should have clearly defined goals and, if necessary, any predetermined hypotheses. The empirical studies included in this review have been observed to have clearly stated aims which were discussed in Section “Research question/aim.”

#### Selection bias

This answers the following questions: “Was the cohort recruited in an acceptable way and was the exposure accurately measured to minimise bias?” The control and intervention groups chosen for comparison are expected to have as many characteristics in common as possible, except for their smoking status. The study population examined by Lee et al. (2018) consisted of 758 men. Women were excluded in this study because the percentage of female smokers recorded from the sample was too low (2.16%). Subjects who had taken cholesterol-lowering medication, antibiotics, or probiotics were excluded because such medications could affect GM. In a similar fashion, the eligibility criteria (Nolan-Kenney et al., 2020) included the absence of antibiotic use by respondents in the previous month and willingness of respondents to provide stool samples at the clinic and answer lifestyle questionnaire. In this study, not much was considered about other factors that could influence the diversity of GM.

Stewart et al. (2018) took cognisance of a couple of factors when recruiting participants. Those who passed the Fagerstrom test for nicotine dependency with a value of ±4 and smoked at least 10 cigarettes daily met the inclusion criteria for tobacco smokers in this study. They stated that there were no significant differences in the sex (6.67% of females), age, diet pattern, height/weight, or race of the subject variables. However, unlike the previous study, it was not clearly stated whether other factors that could affect the diversity of the GM were considered.

Lin et al. (2020) did not include female participants in their study because of the significant gender imbalance between smokers, drinkers, and non-smokers/non-drinkers. Other exclusion criteria included the use of probiotics, antibiotics, or proton-pump inhibitors within the previous month; symptoms of liver, heart, kidney, or lung diseases; diabetes mellitus; or thyroid disease. Others included the presence of digestive tract-related diseases or surgeries, such as gastrointestinal polyp, gastric ulcer, intestinal adenoma, or gastrointestinal tumours.

Also, Yan et al. (2021) carefully outlined exclusion criteria to guarantee that participants were not predisposed to elements that can distort their research findings. Exclusion criteria included (1) any systemic disease (such as hypertension and diabetes); (2) excessive alcohol consumption (more than 25 grams per day for men and more than 15 grams per day for women); (3) use of any of the following medications during the previous month: antivirals, antibiotics, hypoglycaemic medications, blood pressure-lowering medications, lipid-lowering medications, or stomach medications; and (4) an abnormal abdominal ultrasound test.

Ideally, inclusion/exclusion criteria should produce a sample that is representative of the intended general population (Verster et al., 2017). However, in some empirical studies, the ratio of the number of study participants to the number of eligible subjects is usually low. This ratio is referred to as participation rate, and it can indicate the presence of a significant degree of selection bias (Stone et al., 2023). Out of the 1463 eligible subjects approached by Lee et al. (2018), the study participants were made up of 758 men (51.81%). Nolan-Kenney et al. (2020) recorded a high participation rate of 76.22%, while Stewart et al. (2018), Lin et al. (2020), and Yan et al. (2021) did not report the total number of eligible subjects in their studies.

#### Was the outcome accurately measured to minimize bias?

Most intestinal microbiota research uses samples from faeces since they are naturally collected, are non-invasive, and may be collected multiple times (Tang et al., 2020). Although it is asserted that faecal samples cannot serve as indications of the make-up and metagenomic activity of mucosa-associated bacteria dispersed throughout numerous regions of the gut (Zmora et al., 2018), under some practical research conditions, fresh stool samples would sometimes have to be stored for a period before analysis. As a result, the gold standard for GM profiling has been universally accepted as faecal materials since they can be promptly frozen at - 80 °C while preserving microbial integrity without preservatives. This method avoids the potential negative effects of preservatives while preserving microbial components equivalent to those of fresh samples (Fouhy et al., 2015). Due to the above, empirical studies recruited for this review were those that collected samples by extracting DNA from faecal samples using designated DNA extraction kits. Following this protocol, the researchers were able to accurately ascertain the relative abundance of the various genera of intestinal microorganisms in each group. The assessment strategy and results for the risk of bias are presented in Table 5.

#### Have the authors identified and considered all important confounding factors?

Confounding occurs when a relationship between exposure and result is distorted by a different component that is related to both the exposure and the result. Using exclusion criteria, the authors were able to eliminate some confounders, such as systemic disease, excessive alcohol intake, and usage of certain drugs during the preceding month (Stewart et al., 2018). Similarly, Yan et al. (2021) avoided confounders by recruiting participants who met the inclusion and exclusion criteria outlined in the section discussing selection bias. Lee et al. (2018), Lin et al. (2020), and Nolan-Kenney et al. (2020) also considered similar exclusion criteria when recruiting study participants.

#### Internal and external validity or generalizability of the reviewed studies

Internal validity refers to the extent to which research findings accurately represent the population under study and are not the result of methodological defects. The internal validity of a study can be compromised by several factors, including measurement and participant selection errors. This further explains why keen attention was paid to the sampling and measurement protocols of the studies under review. When the internal validity of a study has been established, the researcher can assess its external validity by determining whether the findings hold true for individuals in a different context who are like those in the study. Some strengths recorded in the reviewed studies which could guarantee the generalizability of the findings include the large sample size used, the clear relationship between smoking status and GM, dose–response relationship, and the exclusion criteria that would prevent confounding.

The generic critique identified from the evaluated studies includes the fact that they were majorly cross-sectional studies which cannot determine causality. Secondly, 16S amplicon-based sequencing data were used which can only identify isolates to the genus level except for Yan et al. (2021). Thirdly, most of the reviewed studies had only male participants because there was an insignificant number of eligible female participants in the sampled population. Finally, even though the use of some medications was excluded, there could be effects of potential confounders such as diet and other medications which were not considered in some of the studies.

Although it is recommended that these concerns be considered, it can be argued that the identified criticisms could not have affected the results. Sequencing based on the 16S amplicon, for example, could detect the variation in GM diversity between populations. Similarly, using only male participants yielded valid results because differences in the composition of GM between genders can only be attributed to metabolic disorders and their co-morbidities (Santos-Marcos et al., 2018).

#### Narrative synthesis of results

Intestinal microbiota changes brought on by CS exposure were explored in the reviewed research. At the start of the studies, the baseline characteristics of enrolled subjects were taken to summarize important attributes of the participants enrolled. This includes mean age ranging from 44.2 ± 9.1 to 57.21 ± 17.40 years and body mass index (BMI) ranging from 21.5 ± 4.1 to 24.86 ± 3.50 kg/m^2^. Participants had an average of 2.4 years of formal education, and average muscle and fat mass was 52.8 ± 5.8–52.5 ± 5.4 kg and 17.3 ± 5.7–17.1 ± 4.9 kg, respectively. Targeting a young population was important because in older adults over the age of 70, immunological activity decline, changes in digestion and nutrient absorption, and changes in immune function can all have an impact on the make-up of the GM. Changes in dietary habits (more monotonous) may potentially reduce the variety of the gut bacteria (Rinninella et al., 2019). It is also important to note that BMI levels can predict dysbiosis in the GM. The microbiota of obese individuals, for example, contains low relative proportions of *Bifidobacterium vulgatus* and high concentrations of *Lactobacillus* spp. (Bervoets et al., 2013).

According to the Shannon index of alpha diversity, there were no significant differences in the richness and evenness of the gut microbial taxa among never smokers, former smokers, and current smokers (Lee et al., 2018; Stewart et al., 2018; Lin et al., 2020; Nolan-Kenney et al., 2020). The Shannon index of alpha diversity is a scientific method for assessing the richness and diversity of a sample (Thukral, 2017). Richness is a measure of the number of various species, whereas diversity is a measure of the relative abundance of different species in terms of their evenness of distribution (Willis, 2019). However, Yan et al. (2021) found a significant difference in the alpha diversity of GM between cigarette smokers and non-smokers using a more comprehensive evaluation technique termed WGS. Although there was also no significant difference between non-smokers and former smokers, all investigations found that there were significant differences in the beta diversity indices between people who smoked and those who did not.

Current smokers displayed a higher relative abundance of the phylum Bacteroidetes, a lower relative abundance of the phylum Firmicutes, and a lower Fir/Bac ratio as compared to never smokers (Lee et al., 2018; Stewart et al., 2018). In addition, Lee et al. (2018) reported that the organisms in the intestines of never and current smokers were similar at the family level but distinct at the phylum level. SCFA concentrations and the Fir/Bac ratio of the two major microbial phyla are usually recognized as critical indicators of a person’s gut health condition. Indigestible food components are converted to SCFAs by the healthy gut flora. The gut pH is acidified by SCFAs like acetic, propionic, and butyric acids, which prevent harmful bacteria like Enterobacteriaceae from growing (Ghosh et al., 2011).

Nolan-Kenney et al. (2020) compared current smokers and never smokers and found that the relative abundance of 14 taxa was nominally significantly associated with smoking status. They reported that after accounting for multiple comparisons, present smokers had considerably higher concentrations of bacterial taxa along the Erysipelotrichia-to-Catenibacterium lineage than non-smokers. The odds ratios between the mean relative abundance of present smokers and never smokers were 1.91 for the genus Catenibacterium (false discovery rate (FDR)-adjusted p = .01), 1.89 for the family Erysipelotrichaceae (FDR-adjusted p = .002), 1.89 for the order Erysipelotrichales (FDR-adjusted p = .001), and 1.89 for the class Erysipelotrichia (FDR-adjusted p = .0008). When compared to never smokers, current smokers also had higher concentrations of bacteria from the Coriobacteriia-to-Collinsella lineage, but these differences were not statistically significant.

With the aid of linear discriminant analysis (LDA) effect size (LEfSe) analyses conducted by Yan et al. (2021), 94 species were found to be significantly different between smokers and non-smokers. With a specific emphasis on methods that involve direct recovery of genetic materials from a sample, the LEfSe approach is employed to support high-dimensional class comparisons. By combining traditional tests for statistical significance with additional tests expressing biological consistency and impact relevance, LEfSe discovers the characteristics (operational taxonomic units, genes, or functions) that can appropriately clarify differences between classes.

Fifty-three species were enriched in the smokers, including *Bacteroidales bacterium (B. bacterium)* pH 8, *Bacteroides eggerthii (B. eggerthii), Bacteroides faecis, Bacteroides gallinarum (B. gallinarum), Bacteroides massiliensis (B. massiliensis), Bacteroides salyersiae, Bacteroides stercoris (B. stercoris), Bacteroides vulgatus,* and *Bacteroides xylanisolvens*; *L. bacterium* 1157FAA*, L. bacterium* 2146FAA*, L. bacterium* 3146FAA*, L. bacterium* 3157FAACT1*, L. bacterium* 8157FAA, and *L. bacterium* 9143BFAA*;* and *R. albus, R. bromii, Ruminococcus callidus, Ruminococcus gnavus (R. gnavus), Ruminococcus lactaris, Ruminococcus obeum,* and *R.* sp. 5139BFAA. Forty-one species were enriched in the non-smokers, including *Alistipes finegoldii, Alistipes indistinctus (A. indistinctus), Alistipes onderdonkii, Alistipes putredinis, Alistipes senegalensis (A. senegalensis), Alistipes shahii,* and *A.* sp*. AP11; Bacteroides caccae (B. caccae), Bacteroides cellulosilyticus (B. cellulosilyticus), Bacteroides clarus (B. clarus), Bacteroides intestinalis (B. intestinalis), Bacteroides nordii (B. nordii), Bacteroides oleiciplenus (B. oleiciplenus), Bacteroides plebeius (B. plebeius),* and *Bacteroides uniformis (B. uniformis); Eubacterium eligens (E. eligens), Eubacterium ramulus (E. ramulus), Eubacterium rectale (E. rectale),* and *Eubacterium ventriosum (E. ventriosum);* and *Roseburia hominis (R. hominis), Ruminococcus torques (R. torques),* and *Roseburia inulinivorans (R. inulinivorans)* (Yan et al., 2021).

Yan et al. (2021) further reported that certain organisms enriched in the smokers, including *R. albus*, *R. bromii*, *B. bacterium* pH 8, and *B. eggerthii,* were positively correlated with inflammatory markers. Other bacteria, such as *R. hominis, R. torques, R. inulinivorans, E. eligens, E. ramulus, E. rectale,* and *E. ventriosum,* were negatively correlated with inflammatory markers and were enriched in non-smokers.

In agreement with the findings, Lin et al. (2020) noted that Bacteroidetes, Firmicutes, and Saccharibacteria showed substantial differences in phylum-level abundance. The abundance of Firmicutes was noticeably lower in the smoking/drinking group and the smoking group, while the abundance of Bacteroidetes was higher in the smoking group than it was in the non-smoking/non-drinking group. The relative abundance of Bacteroides, Pseudomonas, and Actinomyces increased in the smoking group and the smoking/drinking group when compared to the non-smoking/non-drinking group, while *R. gnavus* group increased and Phascolarctobacterium declined exclusively in the smoking group. There were no discernible differences between the drinking/smoking group and the smoking group when they were compared to the drinking group; however, Actinomyces increased in the drinking/smoking group.

Results from this study indicate that even after smoking was stopped, the effect of cigarette smoking on the relative abundance of some bacterial species in the gut persisted for some time. Five of the bacterial taxa in the gut that were considerably more abundant or had a larger proportion of presence at the nominal level in current smokers compared to never smokers were also significantly more prevalent in former smokers than in never smokers (Nolan-Kenney et al., 2020). These taxa included class *Alphaproteobacteria*, class *Erysipelotrichia*, order *Erysipelotrichales*, family *Erysipelotrichaceae*, and genus *Slackia.* The effect, albeit continuing after stopping smoking, may deteriorate over time, as evidenced by the relationship between past smokers and never smokers being lower than that between present smokers and never smokers. Former smokers do not exhibit any of the other taxa that were nominally significant when comparing present smokers to never smokers.

## Discussion

### Overview of the findings

Although microbes are present on practically all body surfaces, the gut has the greatest number of microbial communities (Sender et al., 2016). Human GM composition significantly changes because of cigarette smoking exposure (Lee et al., 2018; Stewart et al., 2018; Lin et al., 2020; Nolan-Kenney et al., 2020; Yan et al., 2021). Due to the prevalence of cigarette use and the significance of intestinal microbiota, smoking-induced dysbiosis is a significant public health concern. However, not so much has been done on the relationship between smoking and gastrointestinal microbiota (Antinozzi et al., 2022). Five empirical studies met the inclusion and exclusion criteria of this SLR. These publications were compiled and synthesized to gain a greater understanding of the available evidence which will be useful for policy and practice.

### Exclusion criteria

Besides the age of the respondents, a few other exclusion factors were considered when recruiting the respondents. These exclusion factors included the presence of any systemic disease, excessive alcohol consumption, and abnormal abdominal ultrasound results. Also considered was the use of antibiotics, antivirals, probiotics, hypoglycaemic medications, blood pressure-lowering medications, lipid-lowering medications, or stomach medications during the previous month. Because there are several endogenous and exogenous factors that affect the intestinal microbiota, it is important to minimize confounding variables to avoid skewing the results. Some of these factors identified by researchers include birth method (Kapourchali and Cresci, 2020), diet (Cresci and Bawden, 2015), geographic location (Prideaux et al., 2013), medication (Maier and Typas, 2017), and ailment (Dahiya and Nigam, 2022). To control these variables, the SLR included only empirical studies that recruited healthy participants, and these studies were conducted in various geographic locations.

In the Belgian Flemish Gut Flora Project (FGFP) and Dutch LifeLines DEEP research, medications for diseases that people use daily had the biggest effects on the composition of the microbiota (Falony et al., 2016; Zhernakova et al., 2016). This finding is not unexpected given how non-antibiotic medications affect commensal bacteria: in vitro bacterial growth was reduced by 24% of 1000 popular medicines (Maier et al., 2018). Studies looking at the link between dysbiosis of the GM and T2D have demonstrated the significant confounding effect of medication. Patients with T2D were categorized in a study based on their use of metformin (Forslund et al., 2015). A decrease in butyrate producers was correlated with an increase in Lactobacillus with illness in metformin-naive patients. The therapeutic and unfavourable effects (diarrhoea, bloating) of this most popular anti-diabetic drug, however, may be explained by a large increase in Escherichia with illness in metformin-treated T2D patients. Therefore, to accurately measure GM diversity, studies of the GM must be stratified for medications and other confounding variables. Otherwise, changes in the microbiota can only be the result of these variables (Maier and Typas, 2017; Dahiya and Nigam, 2022).

### Taxonomic characterization of the GM

Rapidly expanding research on the influence of environmental factors on the composition of the gastrointestinal bacterial community has been conducted to evaluate potential links with human diseases and pathologies (Allais et al., 2016). The 16S rRNA gene sequence-based bacterial analysis approach was used by four out of the five studies reviewed. These four studies observed that there were no significant differences in the richness and evenness of the gut microbial taxa among never smokers, former smokers, and current smokers (Lee et al., 2018; Stewart et al., 2018; Lin et al., 2020; Nolan-Kenney et al., 2020).

However, Yan et al., (2021) recorded a significant difference in alpha diversity of GM of cigarette smokers compared to non-smokers using WGS. This is in tandem with the findings of Johnson et al. (2019) and Durazzi et al. (2021), who used silico and sequence-based research to critically re-evaluate the potential of 16S gene to give taxonomic resolution at the species and strain level. Targeting of 16S variable areas using short-read sequencing technologies was shown to be unable to obtain the taxonomic resolution provided by sequencing the whole (1500 bp) gene. This explains why, unlike other researchers, Yan et al. (2021) found a significant difference in the alpha diversity of the evaluated GM.

Yan et al. (2021) further observed that the enriched gut microorganisms in smokers had a positive correlation with inflammatory indicators, whereas the enriched gut microorganisms in non-smokers had a protective effect and a negative correlation with inflammatory markers. The bacteria with the most negative correlation with inflammatory markers and the highest production of SCFAs*, E. ramulus, E. rectale,* and *E. ventriosum*, were concentrated in the non-smokers. Another important bacterium was *Adlercreutzia equolifaciens (A. equolifaciens)*, which was more prevalent in non-smokers. *A. equolifaciens* participates in the metabolism of polyphenols and produces bioactive compounds that can treat metabolic disorders like diabetes and obesity (Clavel et al., 2014).

Non-smokers had higher concentrations of *B. caccae, B. clarus, B. cellulosilyticus, B. intestinalis, B. oleiciplenus, B. nordii, B. plebeius*, and *B. uniformis.* Increased *B. plebeius* in faecal microbiota transplant patients with colitis was linked to illness (Hiippala et al., 2020). Patients with colitis whose *B. plebeius* levels were elevated during faecal microbiota transplantation had illness. To reduce inflammation, *Clostridium leptum* (*C. leptum*) in mice raised the number of regulatory T cells in the spleen (Li et al., 2012) and prevented the production of inflammatory cytokines (He et al., 2020). Non-smokers had higher concentrations of *R. hominis* and *R. inulinivorans.* All these microorganisms create butyrate and SCFAs, which digest polysaccharides and lessen inflammation (Chu et al., 2019; Ticinesi et al., 2020; Zheng et al., 2020).

Racial and ethnic differences are among the criteria used to assess changes in the composition of the human GM, in addition to health and lifestyle (Byrd et al., 2020). One of the primary factors influencing racial and ethnic diversity in the microbiota is historical lifestyles and diet. The research of gut bacterial diversity depending on ethnicity has attracted the most attention in Asian countries, where adults, children, healthy people, and those suffering from a range of illnesses were researched (Xu et al., 2020; Dwiyanto et al., 2021; Takagi et al., 2022). As a result, the microbiota of four Malaysian communities including Malays, the Chinese community, Indians, and one of the country’s indigenous tribes, the Jakun, were examined (Dwiyanto et al., 2021). The dominating taxa included Prevotella, Bacteroides, and Bifidobacterium. A characteristic of the Jakun gut was the identification of *Klebsiella quasipneumoniae*, whereas the Indigenous population and the Chinese population were distinguished by a significant number of Prevotella and Bacteroides. The participants in the empirical research under evaluation were sourced from various parts of the world, and their GM diversity is predicted to be influenced by differences in their ethnic, religious, and cultural lifestyles. Participants came from China, Korea, Bangladesh, and the United States. The GM composition of current smokers differed significantly from that of never smokers, regardless of race, while, between never smokers and former smokers, there was no difference in the composition of the GM.

### Health implication of findings

The function of the microbiota in health and disease has regained interest with the development of culture-independent approaches for characterizing microbial populations. Powerful tools for in-depth investigation of the microbiota have been made available by next-generation sequencing techniques (Le Chatelier et al., 2013; Sheehan and Shanahan, 2017). In an interventional study, various methods were used to detect significant alterations in the faecal microbiota of healthy people quitting smoking. These alterations included an increase in the relative abundance of Actinobacteria (high guanine and cytosine content bacteria, and Bifidobacteria), Firmicutes (*Clostridium coccoides, C. leptum* subgroup, and *E. rectale*), and a decrease in Bac (b- and g-subgroup) (Biedermann et al., 2014). According to a cross-sectional study that used fluorescence in situ hybridization to focus on specific bacterial groups, smoking patients with active Crohn’s disease (CD) displayed distinct microbial profiles with a greater Bacteroides–Prevotella count than non-smoking patients with CD (Benjamin et al., 2012). Recurrent episodes of intestinal inflammation are a defining feature of the IBD known as CD, which can cause serious consequences and disability (Büsch et al., 2014). Similar findings were also observed in non-smoking healthy controls, indicating that the link may not be caused by intestinal inflammation but rather by a direct effect of smoking on the microbiota.

Uncertainty exists over the pathophysiological mechanism by which smoking damages the colon and causes intestinal inflammation such as IBD and ulcerative colitis (UC). Intestinal cytokine levels changing, altered mucosal immune response, and decreased gut permeability have all been hypothesized as ways by which smoking causes intestinal inflammation. Few human studies have revealed that IBD patients have an unbalanced GM in the active period (Sokol and Seksik, 2010; Halfvarson et al., 2017). The intestinal microbiota of IBD patients were discovered to have excessive amounts of *Proteus mirabilis* and *Klebsiella pneumoniae* (Walker et al., 2011; Morgan et al., 2012; Grivennikov, 2013; Zhu et al., 2013; Haberman et al., 2014).

UC represents a form of chronic recurring inflammation specifically affecting the colorectal area and the mucosal lining of the digestive tract (Huang and Shi, 2019). Some research has indicated disturbances in the microbial composition of the gut in UC patients, characterized by reduced taxonomic diversity, declines in Firmicutes, and elevations in Proteobacteria within their gut microbiomes (Huttenhower et al., 2014; Jacobs et al., 2016). The prevalence of the *Fusobacteriaceae* family rose, while Bifidobacteria and constituents of the *Faecalibacterium* taxon seemed to be diminished in the GM of individuals with UC (Reshef et al., 2015; Duranti et al., 2016). Subsequent investigations proposed that the decreased presence of *Bifidobacteria* could serve as a microbial indicator for identifying intestinal dysbiosis associated with the onset of UC (Duranti et al., 2016).

Furthermore, the observed changes in the GM following smoking cessation – increased Actinobacteria and Firmicutes and decreased Bacteroidetes – were comparable to those noticed in obese versus lean humans and mice (Savin et al., 2018). These results raise the possibility that the aetiology of weight gain following smoking cessation, which is typically attributed to dietary changes, may involve smoking-induced intestinal dysbiosis. Dysbiosis brought on by smoking may also contribute to the emergence of illnesses outside the digestive tract. For instance, epidemiological data suggest that smoking is a defence against Parkinson’s disease. According to one theory, smoking alters the microbiota of the intestine in a way that prevents the protein alpha-synuclein from misfolding as much in the enteric nerves. By halting the spread of the protein aggregates in the central nervous system, this may lower the likelihood of developing Parkinson’s disease (Derkinderen et al., 2014).

Also, it is understood that GM contributes to the metabolism of substances that are potentially harmful, nutritive, and therapeutic (Jandhyala et al., 2015; Claus et al., 2016). Smoking cigarettes can cause the body to absorb several harmful chemicals that can alter metabolism and GM make-up. Cigarette smoking, which has been shown to affect microbiota composition, may indirectly affect immune function because microbiota have recently been linked to host immunological function (Thomas et al., 2017).

Lin et al. (2020) discovered a substantial positive association between Bacteroides and smoking pack/year. Bacteroides species are Gram-negative, bile-resistant, anaerobic rods. Although Bacteroides are thought of as carbohydrate processors in the gut to provide energy sources for the cells of the gut epithelium, they are present in most anaerobic infections linked to more than 19% mortality (Wexler, 2007). In the gut, the bacteria typically coexist in stable balance with the host, but when this equilibrium is upset by bacterial overgrowth or host dysfunction, the bacteria may start to pose a threat to the health of the host (Yang et al., 2022). According to Partida-Rodríguez et al. (2017), a substantial Bacteroides population triggers the host’s pathological response and encourages the development of acute abscesses, intestinal blockage, blood vessel erosion, and even fistulas. The ability of Bacteroides to evade the host immune response by preventing macrophage activity and modifying surface polysaccharides is yet another detrimental trait (Hsieh et al., 2020). The pathogenic effects of these bacteria are supported by the increased bacterial toxin pathway in smoking subjects, the positive correlation between the load of Bacteroides and the bacterial toxins, and the elevated level of host carcinoembryonic antigen linked to the load of Bacteroides in this study.

The impact of smoking on the gastrointestinal system has been extensively examined as a potential risk factor for cancer, as noted by Cicchinelli et al. (2023). Commencing with studies using animal models, researchers have observed that mice exposed to smoke exhibited dysbiosis in the GM, leading to an elevated occurrence of CRC. This phenomenon was attributed to heightened pro-tumoral metabolites and compromised gut barrier function, potentially activating oncogenic mitogen-activated protein kinase/extracellular signal-regulated kinase (MAPK/ERK) signalling in the colonic epithelium (Bai et al., 2022).

In human CRC patients, there is an observed increase in the prevalence of *Streptococcus gallolyticus*, *Fusobacterium, Bacteroides fragilis,* and *Escherichia–Shigella,* alongside a depletion of genera such as *Bacteroides, Roseburia,* and *Pseudomonas.* Smoking is a well-established factor implicated in the initiation of CRC. Although the precise mechanisms responsible for the detrimental effects of smoking in CRC require further elucidation, Huang and Shi (2019) have suggested a potential role of ingesting bacteria present in cigarettes.

Additional research has explored the impact of smoke-induced gut dysbiosis on the development of cardiovascular diseases, yielding divergent findings. Hu et al. found a reduction in species affiliated with *Bifidobacteria* and *Akkermansia*, coupled with an increase in *Enterococcus faecium* and *Haemophilus parainfluenzae* among individuals currently smoking and diagnosed with coronary artery disease (CAD), as opposed to those who were former or never smokers (Hu et al., 2021). These alterations led to changes in microbiota-derived metabolites associated with atherosclerosis, and such changes were reversible upon smoking cessation.

### Strengths of the study

The most compelling aspect of this study is that it revealed the connection between GM and smoking status synthesizing results from recent primary studies. The reviewed studies involved many respondents, and these respondents were recruited following exclusion criteria that could lead to confounding and bias of the results. Also, the results of Nolan-Kenney et al. (2020) are consistent with research on how smoking affects the bacterial species richness and diversity in other parts of the body and show a dose–response relationship, supporting the findings that some taxa are more numerous in smokers. In addition, a sizable number of former smokers who were recruited for some of the research can be used to postulate the long-term consequences of quitting smoking on GM. The fact that participants in the numerous empirical investigations were chosen from a variety of geographical backgrounds, which is thought to have an impact on the microbial diversity of the gut (as explained in Section “Taxonomic characterization of the GM”), is one important feature that makes the conclusions of this review robust. Another advantage of this review is its capacity to highlight the relative significance of WGS, which was able to identify a significant difference in the GM alpha diversity between cigarette smokers and non-smokers in contrast to the 16S rRNA approach, which found no differences in the richness and evenness of the GM taxa among former smokers, never smokers, and current smokers according to the Shannon index of alpha diversity. Aside from the number of strengths accredited to this study, it also has a few limitations which are discussed below.

### Limitations

There are certain restrictions on the review. The first is the use of cross-sectional study designs in the examined studies, which cannot establish causality. The second drawback is that most of the research only used 16S rRNA gene sequencing, which has genus-level precision and does not allow for direct functional profiling. To better comprehend these pathways, metagenomic sequencing studies are required to assess how smoking interacts with the gut microbiome. Furthermore, although a variety of confounding factors were noted in the trials, none of them included food, which could be a significant confounder. Finally, most studies only included male participants, while the single study that included female participants had only 2/30 female participants. Further research is required to ascertain whether the findings differ across males and females considering the possibility of sex-specific microbiome profiles (Haro et al., 2016).

### Recommendation for further research

Numerous hypotheses regarding the observed changes in the compositions of bacterial community can be proposed based on the known effects of smoking, such as alteration of the immune system (Sørensen et al., 2010), changes in oxygen tension (Jensen et al., 1991), and direct antibacterial action (Pavia et al., 2000). The GM of non-smokers was much more diverse than that of smokers. Given that changes in immune homeostasis and decreased diversity brought on by smoking may negatively influence disease statuses of smokers in relation to microbe–immune interactions, further research into these interactions is necessary.

These modifications in microbiota composition brought on by smoking may contribute to the aetiology of several disorders because microbiota diversity is generally associated with health (Requena et al., 2018). Further research is needed to better understand the mechanism of bacterial dysbiosis brought on by smoking, how smoking affects the metagenomic composition of the gut microbiome, and whether smoking-related changes to the gut microbiome and/or metagenome can shed light on the disease pathogenesis brought on by smoking.

The participants in some of the empirical studies involved convenience sample of people who had regular check-ups, making them more likely to represent the healthy community, while others practically recruited cohorts of healthy individuals. Questionnaires were used in the research to assess the smoking behaviour of participants, which could lead to an underreporting of their real smoking status. Also, the participants’ living environmental condition (such as passive smoking) was not known, which could have affected the findings and, in turn, the analyses. To fully comprehend how smoking affects the GM, these parameters should be taken into consideration for subsequent research.

In addition, it is crucial to suggest futuristic investigations that would investigate the correlation between the GM in individuals diagnosed with lung cancer and those who smoke. Understanding the interplay between these two factors could provide valuable insights into the potential role of GM in the development and progression of lung cancer among smokers. By comparing the microbial profiles of lung cancer patients who smoke with those who do not, researchers can elucidate whether specific microbial signatures are associated with increased susceptibility to lung cancer in smokers. Furthermore, investigating how alterations in the GM influence lung cancer progression and treatment outcomes in smokers may unveil novel therapeutic targets and personalized intervention strategies aimed at mitigating lung cancer risk.

## Conclusion

The purpose of this review was to synthesize recent data on the effect of CS on GMD in active smokers relative to non-smokers, as well as the resulting public health implications. To find research that addressed how CS alters the composition of GM, a thorough search of CINAHL, Medline, PubMed, and Google Scholar was conducted. The search protocol gave rise to five studies (Lee et al., 2018; Stewart et al., 2018; Lin et al., 2020; Nolan-Kenney et al., 2020; Yan et al., 2021). Results from these studies revealed no appreciable differences between never smokers, former smokers, and current smokers in the alpha diversity of the gut microbial taxa (Lee et al., 2018; Stewart et al., 2018; Lin et al., 2020; Nolan-Kenney et al., 2020). However, Yan et al. (2021) found that utilizing WGS, there was a substantial difference between the alpha diversity of the GM of cigarette users and non-smokers. Although there was also no significant difference between non-smokers and former smokers, all investigations found that there were significant differences in the beta diversity indices between people who smoked and those who did not.

Current smokers displayed a higher relative abundance of the phylum Bacteroidetes, a lower relative abundance of the phylum Firmicutes, and a lower Fir/Bac ratio as compared to never smokers (Lee et al., 2018; Stewart et al., 2018). Lee et al. (2018) further asserted that never and current smokers only differed in taxonomic abundance at the phylum level and did not differ at the family level. Also recorded is the fact that the enriched gut microorganisms in smokers had a positive correlation with inflammatory indicators, whereas the enriched gut microbes in non-smokers had a protective effect and a negative correlation with inflammatory markers. Organisms enriched in the smokers and positively associated with inflammatory markers were *R. albus*, *R. bromii*, *B. bacterium* pH 8, and *B. eggerthii.* Other bacteria, such as *E. eligens, E. ramulus, E. ventriosum, E. rectale, R. hominis, R. torques,* and *R. inulinivorans,* were negatively correlated with inflammatory markers and were enriched in non-smokers.

Results from this study also revealed that even after smoking was stopped, the effect of cigarette smoking on the relative abundance of some bacterial species in the gut persisted for some time. The difference in the diversity of the GM of former smokers and never smokers is minimal when compared to the difference observed between never smokers and current smokers. This suggests that the effect, while lasting after quitting smoking, may diminish with time. The other taxa that were nominally significant when contrasting current smokers to never smokers are not present in former smokers.

The GM can boost the immune system (Thomas et al., 2017), control digestion (Passos and Moraes-Filho, 2017), and lessen the chance of developing inflammatory diseases like cancer and diabetes (Halfvarson et al., 2017; Requena et al., 2018). Dysbiosis of the intestinal microbiota is closely associated with diseases of the GIT and extra GIT (Gupta et al., 2022). Maintaining the equilibrium of the GM is therefore a potential therapeutic approach for illnesses related to smoking. Consequently, policymakers and practitioners can utilize the data from this as a useful tool to design strategies for practice as well as educate the public about the effects of smoking on GM.
